# Novel adjuvant Alum-TLR7 significantly potentiates immune response to glycoconjugate vaccines

**DOI:** 10.1038/srep29063

**Published:** 2016-07-21

**Authors:** Cecilia Buonsanti, Cristiana Balocchi, Carole Harfouche, Federica Corrente, Luisa Galli Stampino, Francesca Mancini, Marta Tontini, Padma Malyala, Simone Bufali, Barbara Baudner, Ennio De Gregorio, Nicholas M. Valiante, Derek T. O’Hagan, Rino Rappuoli, Ugo D’Oro

**Affiliations:** 1 GSK Vaccines S.r.l., Siena, Italy; 2 GSK Vaccines, Cambridge, MA, USA

## Abstract

Although glycoconjugate vaccines are generally very efficacious, there is still a need to improve their efficacy, especially in eliciting a strong primary antibody response. We have recently described a new type of vaccine adjuvant based on a TLR7 agonist adsorbed to alum (Alum-TLR7), which is highly efficacious at enhancing immunogenicity of protein based vaccines. Since no adjuvant has been shown to potentiate the immune response to glycoconjugate vaccines in humans, we investigated if Alum-TLR7 is able to improve immunogenicity of this class of vaccines. We found that in a mouse model Alum-TLR7 greatly improved potency of a CRM_197_-MenC vaccine increasing anti-MenC antibody titers and serum bactericidal activity (SBA) against MenC compared to alum adjuvanted vaccine, especially with a low dose of antigen and already after a single immunization. Alum-TLR7 also drives antibody response towards Th1 isotypes. This adjuvant was also able to increase immunogenicity of all polysaccharides of a multicomponent glycoconjugate vaccine CRM_197_-MenACWY. Furthermore, we found that Alum-TLR7 increases anti-polysaccharide immune response even in the presence of a prior immune response against the carrier protein. Finally, we demonstrate that Alum-TLR7 adjuvant effect requires a functional TLR7. Taken together, our data support the use of Alum-TLR7 as adjuvant for glycoconjugate vaccines.

Polysaccharides antigens are T cell independent antigens that can stimulate B cells but are unable to generate B cell memory and isotype class switching. Glycoconjugate vaccines are by far more efficacious than capsular polysaccharide vaccines in inducing immune responses^1^. The carrier protein that is covalently linked to the polysaccharide, is able to engage T follicular helper cells that provide help for B cells to produce IgG antibodies against the polysaccharide component, triggering, therefore, a T cell dependent immune response to the polysaccharide. Consequently, glycoconjugates induce polysaccharide-specific IgM-to-IgG switching, long-lived memory B cell development and T cell memory.

Glycoconjugate vaccines are among the safest and most efficacious vaccines developed during the last 30 years. They have played an important role in preventing life-threatening bacterial infectious diseases caused by virulent pathogens such as *Haemophilus influenzae*, *Streptococcus pneumoniae* and *Neisseria meningitidis*^2^. However, the immunogenicity of these glycoconjugates can be influenced by many variables^3^. The type and extent of immune response have to be evaluated on a case to case basis and may strongly depend on the immune-dominance of one vaccine or serotype over the other co-administered vaccines or serotypes. Disadvantages of these vaccines include the need for multiple doses to achieve protective antibody levels and high cost, which makes their use in the developing world problematic. In addition, problems persist in the elderly or immunocompromised individuals, in which immunogenicity has been relatively poor^4^.

Thus, the challenges for further improvement of glycoconjugate vaccines are to identify vaccine formulations and strategies to elicit stronger primary antibody responses in order to achieve serum levels of protective antibodies already after one single immunization and prolonged vaccine efficiency.

Adjuvants are molecules that boost the immune system in order to increase immunogenicity of the vaccine antigens. They may significantly reduce the amount of antigen needed or the number of doses required for optimal response^5^. In addition, they may induce a more rapid response or change the quality of the immune response, leading to increased and broader protection^1^,^6^.

Very few vaccine adjuvants were licensed for human use until few years ago, among which aluminium salts (hydroxide or phosphate), that have been used for many decades, and the oil in water emulsions MF59 and AS03^7^,^8^. Targeting Toll-like receptors (TLRs) is a new and promising approach in the design of new adjuvants. The TLR4 agonist monophosphoryl lipid A (MPL) combined with Aluminium Hydroxide makes indeed the adjuvant AS04, which is contained in an HPV vaccine lately licensed in US and Europe^9^,^10^ as well as in an HBV vaccine^11^.

TLR7 has also been considered as a target for vaccine adjuvant. Indeed, in the paper from Wu. and co-workers^12^ we have recently described a new vaccine adjuvant (Alum-TLR7) based on a TLR7 agonist (SMIP7.10), selected from a benzonaphthyridines series of TLR7 agonists, adsorbed to Aluminium Hydroxide. A relevant proof of concept of Alum-TLR7 adjuvant activity was obtained in combination with a recombinant candidate vaccine against the *Neisseria meningitidis* B (MenB) tested *in vivo* in a relevant animal model, showing a significant increase in functional antibodies against 17 MenB strains leading to a great increase of breadth of coverage when compared to aluminium-adiuvanted vaccine alone. Moreover, we showed that immunization with the Alum-TLR7 formulated recombinant anthrax vaccine leads to rapid priming of naïve T and B cells that is sufficient to provide protection from lethal challenge with *B. anthracis.* No toxicity signs were observed neither systemically nor at the site of injection.

Experience in human on licensed and experimental vaccines have shown that it is very difficult to potentiate the immune response of glycoconjugates by an adjuvant, in particular in primed or pre-exposed adolescents and adults^13^.Therefore, we decided to investigate if Alum-TLR7 is also an efficient adjuvant for glycoconjugate vaccines and examined its adjuvant effect against glycoconjugate antigens of different strains of *Neisseria meningitidis.*

Meningococcal disease worldwide is predominantly a disease of infants and young children. In Europe, *N. meningitidis* serogroup C (MenC) is one of the major serogroups causing invasive disease^14^. Prevention of invasive disease is based on vaccination, with conjugated polysaccharide vaccines being the current standard. The MenC-CRM_197_ conjugate vaccine *Menjugate* (GSK) comprises meningococcal C oligosaccharides conjugated to the protein carrier CRM_197_, a nontoxic mutant of diphtheria toxin (DT). *Menjugate* has been shown to be safe and immunogenic, and is able to prime infants, toddlers, young children and adults for immunological memory. Although the MenC-CRM_197_ conjugate vaccine represents an example of how vaccination with a well characterized antigen can yield pivotal public health triumphs, a need for further improvements, which might yield an increase in the magnitude or breadth of the Men C antigen-specific immune responses, still remains.

We have also considered the case of the quadrivalent glycoconjugate meningococcal vaccine consisting of the four serogroups A, C, W_135_, Y (hereafter MenACWY), aiming to enhance the immune response to the A antigenic component (MenA) which immunogenicity is partly reduced when combined with the C, W_135_ and Y antigenic components in the mouse animal model.

Overall, in this work we analyzed the ability of the new adjuvant Alum-TLR7 to enhance the immune response to MenC-CRM_197_ as a single vaccine component as well as in combination with other glycoconjugate antigens, compared to Aluminium Hydroxide-adjuvanted vaccine alone and we provided the proof of concept that Alum-TLR7 is a promising powerful adjuvant for glycoconjugate vaccines.

## Results

### Alum-TLR7 increases immunogenicity of MenC-CRM_197_ already after one immunization and shifts the response toward a Th1 phenotype

We first evaluated in mice if Alum-TLR7 could enhance and modify the quality of the immune response to the glycoconjugate vaccine MenC-CRM_197_.

Balb/C mice were immunized intramuscularly on days 1 and 28, with a MenC-CRM_197_ based vaccine adjuvanted with only Aluminium Hydroxide (200 μg dose) or Alum-TLR7 with constant 200 μg dose of Aluminium Hydroxide and different doses of the TLR7 agonist as described in material and methods. Both anti-MenC polysaccaride IgG titers and serum bactericidal assay (SBA) titers against the MenC strain of *N. meningitidis* were measured. SBA is a measurement for the induction of functional antibody response, which is critical for protection against infection by *N. meningitidis.*

**Figure 1:**
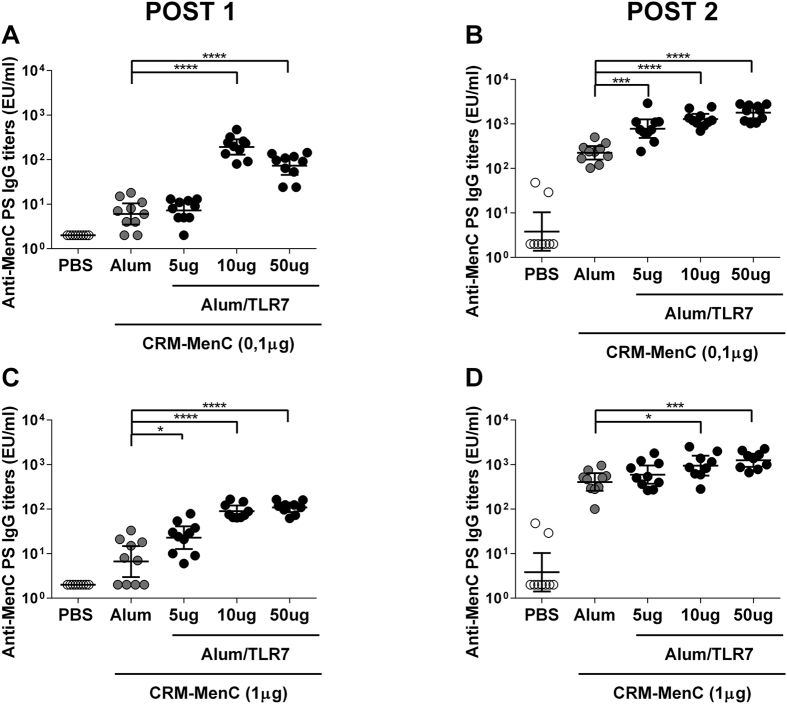
Effect of Alum-TLR7 on the immune response to low doses (**A,B**) and high doses (**C**,**D**) of MenC-CRM_197_ vaccine. Balb/C mice (10/group) were immunized twice, at day 1 and 28, with MenC-CRM_197_ glycoconjugate vaccine antigen alone or formulated with Aluminium Hydroxide or Alum-TLR7 at the indicated doses. 14 days after the first (**A,C**) and the second (**B,D**) immunization, sera were collected and total IgG specific for the MenC polysaccharide were measured by ELISA. Values for each mouse in all groups and the geometric mean (bar) with 95% CI are reported in the graph. Mann Whitney test was applied: ****P < 0.0001, ***P < 0.001, *P < 0.05.

Immunization with a lower concentration of MenC-CRM_197_ (0.1 μg) adjuvanted with higher concentrations of Alum-TLR7 (50 and 10 μg) elicited higher IgG titers against the MenC polysaccharide after one immunization (Fig. 1A) compared to MenC-CRM_197_ adjuvanted with Aluminium Hydroxide. The increased MenC-specific IgG response was observed at all doses of Alum-TLR7 after the second immunization (Fig. 1B). Moreover, the SBA titers against a serogroup C strain were higher in all groups immunized with MenC-CRM_197_/Alum-TLR7 compared to MenC-CRM_197_/Aluminium Hydroxide, after one or two immunizations (Table 1).

A significant increase in IgG titres against MenC polysaccharide was also seen after 1 immunization with a higher dose (1 μg) of MenC-CRM_197_ antigen adjuvanted with Alum-TLR7 (Fig. 1C). An increased anti-MenC IgG response with the Alum-TLR7 adjuvanted formulation was also observed 2 weeks post-2^nd^ dose, although a statistically significant difference was seen only with the higher doses of TLR7 agonist (Fig. 1D). SBA titres against a serogroup C strain were also increased after 1 or 2 immunizations by addition of Alum-TLR7 (Table 1).

**Figure 2:**
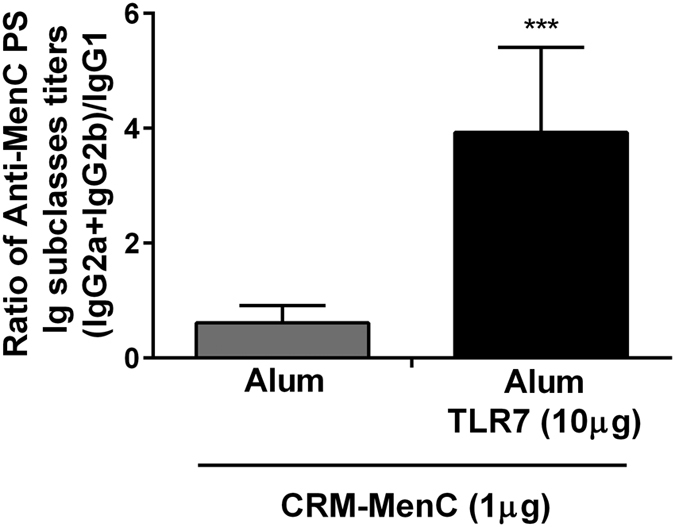
Alum-TLR7 shifts the anti-MenC response to IgG2a and IgG2b (Th1-type). Sera of mice (10/group) immunized twice, 4 weeks apart, with MenC-CRM_197_/Aluminium Hydroxide or MenC-CRM_197_/Alum-TLR7 were collected two weeks after the second immunization and analyzed for immunoglobulin subclasses IgG1, IgG2a and IgG2b. The average ± standard deviation of the ratio between IgG2a+IgG2b and IgG1 of each mouse in the two groups is reported.

In addition to enhancing immune responses as measured by ELISA and SBA, formulating MenC-CRM_197_ with Alum-TLR7 shifted the immune response to a Th1 phenotype as indicated by an increase of the IgG2a+IgG2b/IgG1 ratio in the MenC specific antibody shown in Fig. 2^ ^15.

### Alum-TLR7 induces persistently higher SBA and Ab titers

**Figure 3:**
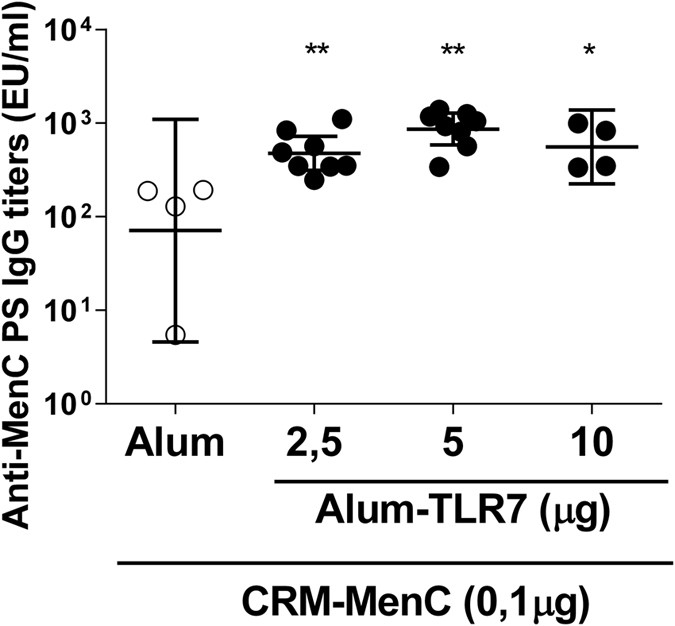
Alum-TLR7 induces persistently higher SBA and Ab titers. 4 or 8 Balb/c mice per group were immunized two times, 4 weeks apart with MenC-CRM_197_/Aluminium Hydroxide or MenC-CRM_197_/Alum-TLR7 at three different concentrations as indicated. Serum anti-MenC antibody titers and SBA titers were measured in the sera 8 months after the last immunizations. Total IgG titers for each mouse in all groups and the geometric mean (bar) with 95% CI are reported in the graph. Mann Whitney test was applied: **P < 0.01; *P < 0.05.

Having established that Alum-TLR7 was able to potentiate the immune response against MenC polysaccharide two weeks after the last immunization, we wanted to investigate if this adjuvant effect was persistent with time analyzing the immune response at later time points after immunization. Therefore we immunized animals twice intramuscularly with a lower dose of MenC-CRM_197_ (0.1 μg) adjuvanted with Alum-TLR7, specifically Aluminum Hydroxide at 200 μg dose and increasing doses (2.5, 5 and 10 μg respectively) of the TLR7 agonist, and measured polysaccharide specific IgG and SBA titers 8 months after the last immunization. At all concentrations of TLR7 agonist, anti MenC polysaccharide IgG titers and SBA titers were significantly higher when compared to the values measured in the sera of mice immunized with MenC-CRM_197_ adjuvanted with Aluminium Hydroxide alone (Fig. 3 and Table 2).

### Alum-TLR7 increases immunogenicity of all glycoconjugate antigens of different *N. meningitidis* strains in a multicomponent vaccine

In the context of vaccines such as meningococcal or pneumococcal vaccines, it is widely accepted to combine multiple serotypes in one single formulation and the current strategy is to develop even more complex combination vaccines to further reduce the number of injections. This approach in turn may compromise the expected immune response to each single antigen which could therefore be improved by addition of an appropriate adjuvant. We considered the case of *Menveo* (GSK), a quadrivalent glycoconjugate meningococcal vaccine consisting of the four serogroups A, C, W_135_, and Y, which is able to stimulate a protective immune response against infection from all these bacterial strains, and evaluated the capacity of Alum-TLR7 to enhance the immune response versus all components and in particular to the A antigenic component (MenA) whose immunogenicity is partly reduced when combined with the C, W_135_ and Y antigenic components in the mouse animal model.

**Figure 4:**
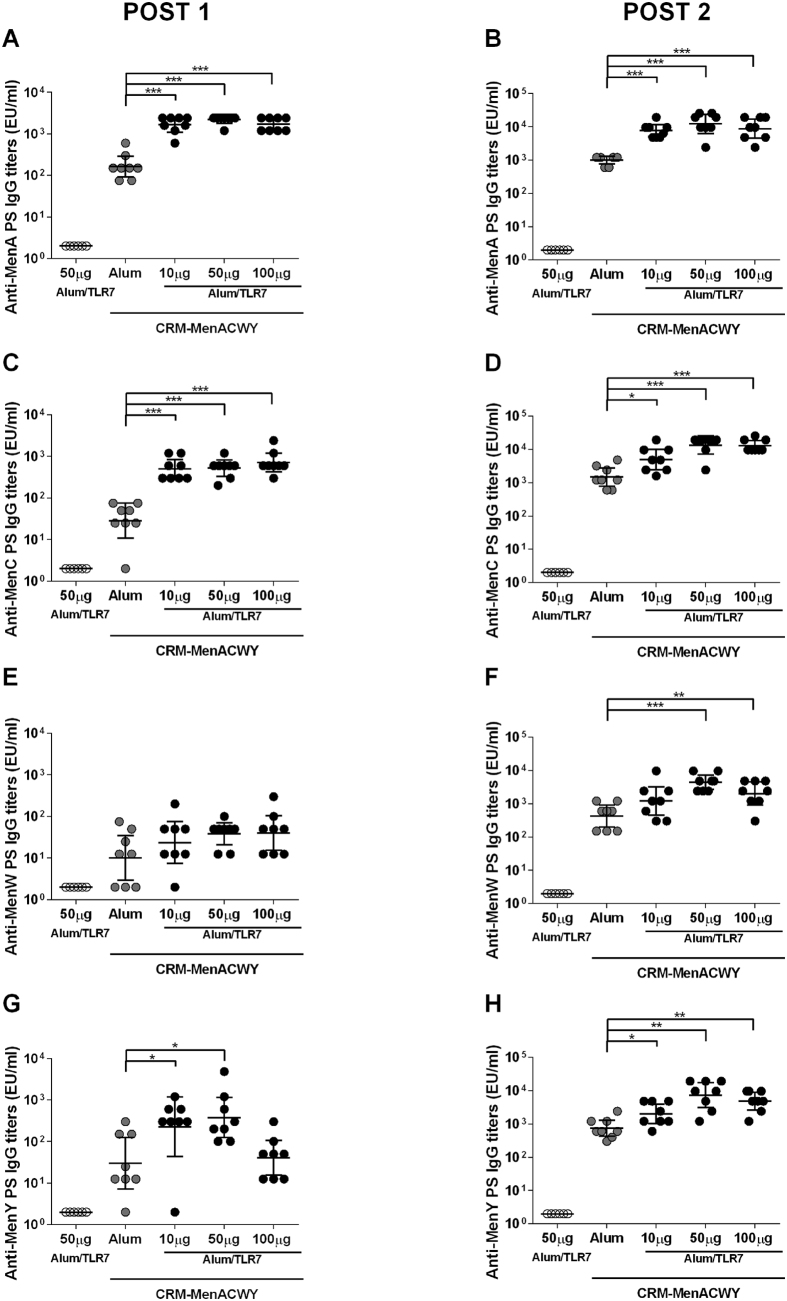
Effect of Alum-TLR7 on the immune response to MenA, MenC, MenW and MenY vaccine antigens. Balb/C mice (8/group) were immunized twice, at day 1 and 14, with MenACWY-CRM_197_ glycoconjugate vaccine antigens formulated with Aluminium Hydroxide or Alum-TLR7 at the indicated doses. 13 days after the first (**A,C,E,G**) and the second (**B,D,F,H**) immunization, sera were collected and total IgG specific for the MenA (**A,B**), MenC (**C,D**), MenW (**E,F**), MenY (**G,H**) polysaccharides were measured by ELISA. Values for each mouse in all groups and the geometric mean (bar) with 95% CI are reported in the graph. Mann Whitney test was applied: ***P < 0.001; **P < 0.01; *P < 0.05.

Balb/C mice were immunized intramuscularly with the MenACWY vaccine formulated with Aluminium Hydroxide or Alum-TLR7 with different doses (10, 50, 100 μg) of TLR7 agonist as described in material and methods, at days 1 and 14. Sera were collected 13 days after each immunization to analyze specific IgG titers, using ELISA, and to perform SBA against each of the vaccine polysaccharide antigens. Compared to Aluminium Hydroxide-based formulations, Alum-TLR7 adjuvanted formulations at all concentrations of TLR7 agonist were able to significantly increase the IgG titers against MenA and MenC polysaccharide after the first and the second immunization (Fig. 4A–D). Moreover, SBA titers against serogroup A and C strains were higher in all groups with Alum-TLR7 compared to Aluminium Hydroxide formulation (Table 3).

When the anti-MenW response was analyzed, the increase in IgG specific titers by Alum-TLR7 after 1 immunization was not significant (Fig. 4E) but an increase in SBA titers against a serogroup MenW strain was observed with the higher doses of TLR7 agonist (Table 3). However, after 2 immunizations the increase of anti-MenW IgG titers in the sera from mice immunized with Alum-TLR7 at higher doses became significant (Fig. 4F) and was associated to an increase in SBA titers at all doses of Alum-TLR7 (Table 3).

Finally, the immune response against the fourth antigen in the vaccine MenY was evaluated. After 1 immunization Alum-TLR7 at both 10 and 50 μg increased anti-MenY IgG titers (Fig. 4G), and higher SBA titers against serogroup Y strain were observed in pooled sera from the three Alum-TLR7 groups (Table 3). A significant increase in anti-MenY IgG titers was observed after 2 immunizations with Alum-TLR7 at all doses (Fig. 4H). An analogous positive effect was induced by Alum-TLR7 after 2 immunizations on the SBA titers against serogroup Y strain (Table 3).

### Alum-TLR7 enhances response to glycoconjugate vaccine in the presence of anti-carrier pre-existing immunity

In order to resemble the human situation, in which the target population of a CRM_197_ based glycoconjugate vaccine is likely to already have a memory immune response against the carrier protein CRM_197,_ due to previous immunizations with DT vaccine or other glycocongugate vaccines, we evaluated the adjuvanticity of Alum-TLR7 for a CRM_197_ based glycoconjugate vaccine in DT-pre-immune mice. Mice previously immunized with a TdaP vaccine (containing DT) or naïve mice were immunized with MenC-CRM_197_ formulated with either Aluminium Hydroxide or Alum-TLR7 with 10 μg dose of the TLR7 agonist. In these conditions, we could investigate if a pre-existing immunity against the carrier protein affected the ability of Alum-TLR7 to increase the immunogenicity of the MenC-CRM_197_ vaccine.

**Figure 5:**
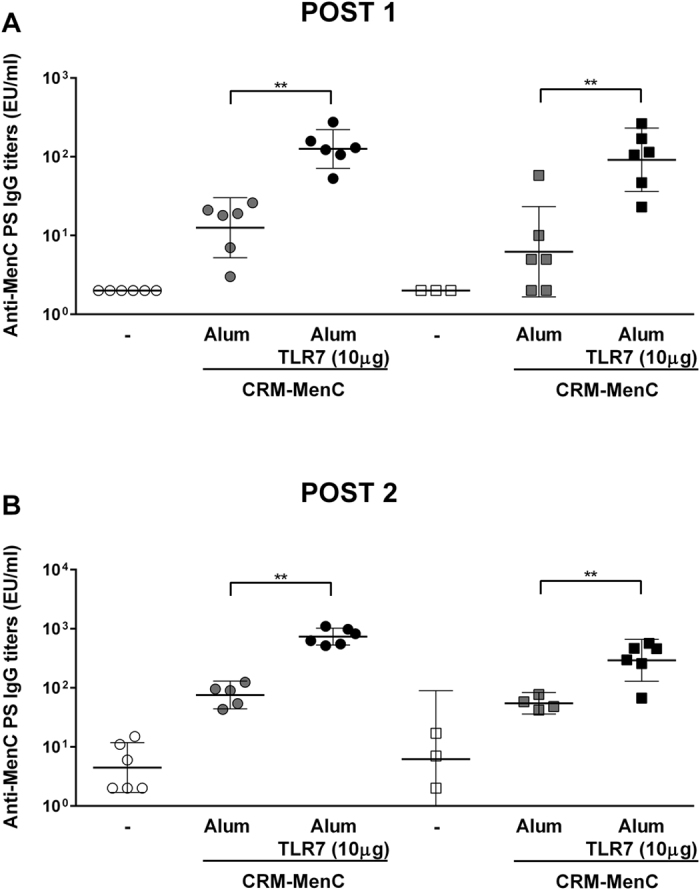
Alum-TLR7 increases the anti-MenC immune response in DT immune mice. Naive mice (dots) or mice that had previously been vaccinated with TdaP antigens (squares), were immunized at day 1 and 28 with MenC-CRM_197_/Aluminium Hydroxide or MenC-CRM_197_/Alum-TLR7. 14 days after the first (**A**) and the second (**B**) immunization, sera were collected and total IgG specific for the MenC polysaccharide were measured by ELISA. Values for each mouse in all groups and the geometric mean (bar) with 95% CI are reported in the graph. Mann Whitney test was applied: **P < 0.01; *P < 0.02.

Similar to naïve mice, immunization with MenC-CRM_197_/Alum-TLR7 elicited higher MenC antibody titers and SBA titers than immunization with MenC-CRM_197_/Aluminium Hydroxide in DT-pre-immune mice after 1 or 2 doses (Fig. 5 and Table 4) demonstrating that the presence of a pre-existing immunity against the carrier protein has no negative effect on the adjuvant potency of Alum-TLR7.

### The adjuvant effect of Alum-TLR7 requires functional TLR7

To evaluate if the adjuvant effect of Alum-TLR7 is mediated by triggering of TLR7 we took advantage of the TLR7-loss-of function mutant mice TLR7^*rsq1*^. In these mice, a point mutation in the amino terminus of TLR7 abolishes signaling without affecting ligand binding^16^.

**Figure 6:**
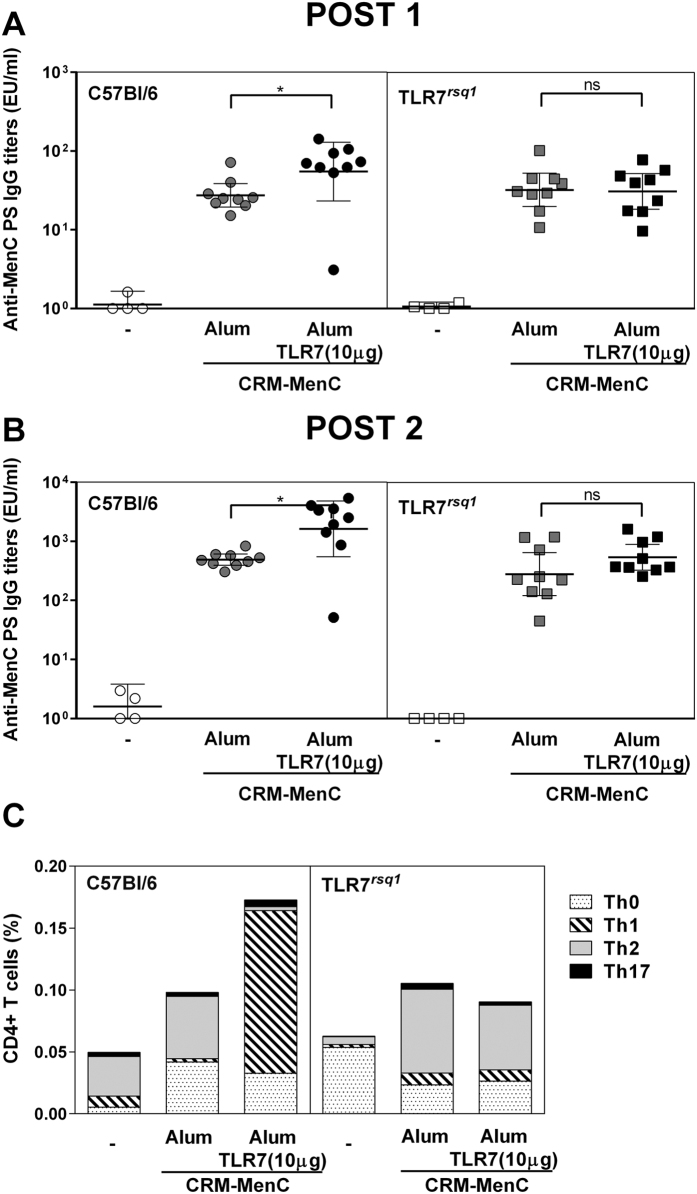
The adjuvant effect of Alum-TLR7 requires functional TLR7. Nine mice per group were immunized at day 1 and 28 with MenC-CRM_197_/Aluminium Hydroxide or MenC-CRM_197_/Alum-TLR7. 21 days after the first (**A**) and 14 days after the second (**B**) immunization, sera were collected and total IgG specific for the MenC polysaccharide were measured by ELISA. Values for each mouse in all groups and the geometric mean (bar) with 95% CI are reported in the graph. Mann Whitney test was applied: *P < 0.01. (**C**) 14 days after the second immunization, splenocytes were stimulated *in vitro* with CRM_197_ and the percentages of Ag-specific cytokine-secreting CD4^+^ T cells were analyzed by intracellular staining. Bars show the average of 9 mice per group of CD4^+^ T cells producing IL-17 with/without IL-2 and TNFα (Th17); IL-2 and/or TNFα (Th0); INF-γ with/without IL-2 and TNFα (Th1); IL-4+IL-13 with/without IL-2 and TNFα (Th2).

We immunized TLR7^*rsq1*^ mice and wild type C57Bl/6 mice with MenC-CRM_197_ (0.1 μg) adjuvanted with Aluminium Hydroxide or Alum-TLR7 with 10 μg dose of the TLR7 agonist. As expected, in wild type mice Alum-TLR7 induced higher anti MenC polysaccaride IgG titers both after the first and the second immunization compared to mice immunized with MenC-CRM_197_ adjuvanted with Aluminium Hydroxide. Interestingly, this effect was completely abrogated in TLR7^*rsq1*^ mice, suggesting that the adjuvant effect of Alum-TLR7 is mediated by the signaling of TLR7 (Fig. 6A,B).

We then wanted to establish if TLR7 signaling was responsible to enhancing isotype switching as revealed by the increased antigen specific IgG2a and IgG2b shown in Fig. 2 that indicates a potent Th1 induction^15^. We therefore analyzed the cytokine expression profile of CRM_197_-specific-CD4^+^T helper cells, re-stimulated *in vitro* with the carrier protein after isolation of the splenocytes from MenC-CRM_197_/Alum-TLR7- or MenC-CRM_197_/Aluminium Hydroxide-immunizated TLR7^*rsq1*^ mice and wild type C57Bl/6 mice. In wild type mice, addition of TLR7 agonist decreased the production of Th2-like cytokines such as IL4 and IL13, and increased the expression of Th1-like cytokines such as IFNγ, determining a switch in the type of the T helper cell response. On the contrary, in TLR7^*rsq1*^ mice the cytokine expression profile was identical, regardless of the presence of TLR7 agonist (Fig. 6C).

## Discussion

Glycoconjugate vaccines are the first example of rational design of vaccines since they couldn’t be obtained by simply growing the microorganism and isolating one of its components. Indeed, the highly immunogenic polysaccharides that coat most pathogens need to be chemically linked to a carrier protein in order to fully activate the adaptive immune system. Even though glycoconjugate vaccines have prevented many life threatening diseases during the last 30 years, several problems still limit the efficacy of these vaccines. For example, most glycoconjugate vaccines require few booster doses to obtain full protection and they are not able to quickly induce a protective anti polysaccharide Ab threshold, which would be critical to promptly face possible infectious diseases outbreaks (Es. Meningitis) and to provide earlier protection to infants. Therefore, improving the efficacy of glycoconjugate vaccines, in particular increasing the rapidity by which they induce a protective immune response, would be a great benefit for public health. Here we describe for the first time a new approach to enhance immunogenicity of a glycoconjugate vaccine by formulation with the new adjuvant Alum-TLR7 showing that, in an animal model, this strategy was able to clearly potentiate the efficacy of both a monovalent and a tetravalent anti-meningococcal vaccine. Indeed, in the sera of mice immunized with Alum-TLR7 we measured higher SBA titers, a parameter that evaluate the functional activity of the antibodies and predicts vaccine effectiveness against meningococcal disease. We still don’t know if this effect will be reproduced in human subjects where most of the adjuvants tested so far have failed to boost immunogenicity of glycoconjugate vaccines. It was previously described that, in a clinical trial, the TLR9 agonist CpG 7909 increased the number of high responders upon pneumococcal immunization within a HIV-infected population^17^, but this effect could be limited to these immunocompromised subjects that usually responds very poorly to pneumococcal immunization compared to healthy individuals.

Also, it will be interesting to perform clinical studies in humans to directly compare the effectiveness with glycoconjugate vaccine of Alum-TLR7 to that of other combination adjuvants, such as AS04 which contains a TLR4 agonist in association to Alum. Indeed, a side by side comparison between a TLR7 and a TLR4-based vaccine adjuvant in mice may be un-predictive of the human clinical response because of differences in the signaling pathway and expression of these two receptors in humans and mice, in particular for the fact that only in mice TLR4 is also expressed on B cells, a condition that may have a big impact on the effect of a TLR4 based adjuvant in this species.

Applying a rational design approach, we have optimized the low molecular weight compound SMIP7.10, for use as a flexible vaccine adjuvant. We hypothesized that the key to the successful use of a small molecule immune-potentiator (SMIP) as vaccine adjuvants was keeping this compound localized at the site of injection, which could have two benefits: 1. Localization would optimize triggering of the desired innate immune responses at the site where the vaccine antigens are concentrated facilitating the initiation of the adaptive immune response to these antigens, and 2. Localization would also reduce the release of the SMIP into the systemic circulation, potentially keeping its serum levels below the concentration that triggers a systemic inflammatory response^18^,^19^,^20^. In fact, while serum cytokine levels were high in mice immunized with the free SMIP7.10, cytokines levels comparable to Aluminium Hydroxyde alone were induced by the same compound when it was adsorbed to Aluminium Hydroxyde^12^.

To achieve this localization, TLR7 agonist SMIPs were engineered allowing them to be adsorbed to Aluminium Hydroxide, a very well-known adjuvant with a long history of safe use in humans. The new formulation Alum-TLR7, made by SMIP7.10 adsorbed to Aluminium Hydroxide was selected for further development as a vaccine adjuvant. It exploits the flexibility of Alum formulation permitting it to be applied to many vaccine candidates. Indeed, in this work we showed that Alum-TLR7 was able to increase the immunogenicity of the existing vaccine *Menjugate* as well as that of the multicomponent vaccine *Menveo*. While Aluminium Hydroxyde generally induces a Th2-biased response, we have shown that Alum-TLR7 induces a Th1 response. It is possible that SMIP7.10 targets a specific DC subset that primes T cell differentiation into a Th1-type. Release of Th1 cytokines from T helper cells, in turn, results in IgG2a and IgG2b antibody switching. Among IgG subclasses, IgG2a and 2b are generally considered to be the most potent for activating antimicrobial effector responses since their Fc regions mediate many effector functions such as antibody-dependent cellular cytotoxicity (ADCC) and complement-dependent cytotoxicity (CDC) that ultimately lead to an increase in bactericidal activity. Moreover, a TLR7 agonist may also induce the production of IL-17 from antigen-specific T helper cells that then contributes in activating antimicrobial activities of neutrophils and macrophages^21^,^22^. Of note, newborn and infants display an impaired Th1 response, which could be responsible for the lower response to vaccines as well as the increased susceptibility to infections in this age population^23^,^24^. Therefore, if Alum-TLR7 would be able to drive a Th1-polarizing response in infants, this could be a great benefit. Another key characteristic of the immune response which influences the efficacy of a vaccine is the persistence of the antibody response. While classical glycoconjugate vaccines might display a relatively short duration of the immune response, thus requiring multiple dosing, we showed that, compared to Aluminium Hydroxide, Alum-TLR7 was able to induce significantly higher protective antibody titers at least up to 8 months from the last immunization, suggesting that this adjuvant could be potentially able to induce a long lasting immune response reducing the requirement for additional boosting doses.

In glycoconjugate vaccines, carrier proteins have the important role of stimulating helper T lymphocytes to elicit an immune response leading to specific, high affinity and long lasting antibody response^3^. Currently, many glycoconjugate vaccines are licensed in US, EU or WHO, and they have CRM_197_, DT or Tetanus toxoid (TT) as carrier proteins. In some cases, a pre-existing immunity against the carrier protein might induce negative interference with the immune response against the polysaccharide antigen^25^. We therefore investigated if a pre-existing immunity against DT could abolish the adjuvant effect of Alum-TLR7 in MenC-CRM_197_ glycoconjugate vaccine. Our data demonstrate that a pre-existing immunity against DT does not interfere with the capacity of Alum-TLR7 to increase the immune response of a glycoconjugate vaccine containing the DT-derived protein CRM_197_, suggesting that this adjuvant could be effective at improving the potency of glycoconjugate vaccines also in an adult/old human population, that is most likely primed with common carrier proteins such as DT or CRM_197_.

Using TLR7^*rsq1*^ mice, we also demonstrated that the adjuvant effect of Alum-TLR7 is completely dependent from signaling of TLR7. However, which cell type expressing TLR7 is involved in the potentiation of the immune response is currently under investigation. These finding together with the observation that microarray analysis of splenocytes from TLR7-KO mice treated *in vitro* with benzonaphtyridine TLR7 agonists does not reveal any gene activation (EDG and UD, personal communication), indicate that Alum-TLR7 has no off target effect and suggest that this adjuvant has an optimal safety profile.

While our manuscript and previously published work have demonstrated efficacy of Alum-TLR7 adjuvant on the antibody response as well as on T helper cells response^21^, additional studies are still required to evaluate any possible effect of this new adjuvant on the T cytotoxic response.

In conclusion, the data presented in our manuscript suggest that Alum-TLR7 is a very promising adjuvant for glycoconjugate vaccines and support its use in human to improve the immunogenicity profile of this class of vaccine.

## Methods

### Animal studies

Groups of 6–10 female 8-week-old Balb/C and C57Bl/6 mice (Charles River) or TLR7^rsq1^ mice were used for *in vivo* experiments. For animal experiments with MenACWY-CRM_197_ conjugate vaccine, animals were immunized intramuscularly at days 0 and 14 with 2 μg of MenA antigen and 1 μg of MenC, MenW and MenY in terms of saccharides content. For animal experiments with MenC-CRM_197_ vaccine, animals were immunized intramuscularly at days 0 and 28 with 0.1 or 1 μg of MenC antigen. Animals were immunized with a total volume of 100 μl (50 μl/leg). Aluminium Hydroxide or Alum-TLR7 was added to the formulations. Briefly, MenA, MenC, MenW and MenY antigens were adsorbed onto 2 mg/ml of Aluminium Hydroxide suspension to obtain a final formulation containing the indicated dose of antigen and 200 μg of Aluminium Hydroxide in 100 μl injection volume. When required, different amounts of TLR7 agonist SMIP7.10 were added to the formulations to obtain the indicated doses of the TLR7 agonist in 100 μl injection volume absorbed to a constant 200 μg dose of Aluminium Hydroxide. Blood samples were collected at 2 weeks following the first and the second immunization for ELISA and SBA assays. In one experiment, mice were immunized with TdaP vaccine adjuvanted with Aluminium Hydroxide three times, two weeks apart, 12 months prior to MenC-CRM_197_ immunization. As control, 12-months old, naive mice (Charles River) were used. All animal studies were approved by GSK Animal Welfare Body and carried out in accordance with current Italian legislation on the care and use of animals in experimentation (Legislative Decree 26/2014) and with the GSK Animal Welfare Policy and Standards.

### Measurements of SBA titers against *Neisseria meningitides*

SBA against *N. meningitidis* strains was evaluated with pooled baby rabbit serum used as complement source. Briefly, *N. meningitidis* reference strains (F8238 for MenA, C11 for MenC, 240070 for MenW_135_ and 860800 for MenY) were grown overnight on chocolate agar plates at 37 °C in 5% CO_2_. Colonies were inoculated in Mueller-Hinton broth, containing 0.25% glucose, to reach an OD_600_ of 0.05–0.08 and incubated at 37 °C with shaking until the OD_600_ reached the value of 0.24–0.26.

Bacteria were diluted in the assay buffer (Gey’s balanced salt solution, GBSS, with 1% BSA w/v for MenACWY SBA) at the working dilution of 10^4 ^CFU/mL. All sera to test were heat inactivated for 30 minutes at 56 °C. The total volume in each well was 50 μL with 25 μL of serial two-fold dilutions of the test serum, 12.5 μL of bacteria at the working dilution and 12.5 μL of baby rabbit complement.

Negative controls included bacteria incubated, separately, with the complement serum without the test serum and with test immune sera and heat inactivated complement.

Immediately after the addition of the baby rabbit complement, negative controls were plated on Mueller-Hinton agar plates using the tilt method (time 0). The plate was incubated for 1 hour at 37 °C, then each sample was spotted in duplicate on Mueller-Hinton agar plates and controls were plated on Mueller-Hinton agar plates using the tilt method (time 1). Agar plates were incubated for 16–18 hours at 37 °C and the colonies corresponding to time 0 and time 1 (surviving bacteria) were counted.

Serum bactericidal titers were defined as reciprocal of the serum dilution resulting in 50% decrease in colony forming units (CFU) per mL after 60 min incubation of bacteria in the reaction mixture, compared to control CFU per mL at time 0. Typically, bacteria incubated without serum in the presence of complement (negative control) showed a 150 to 200% increase in CFU/mL during the 60 min incubation time.

### ELISA assays for antibody against MenACWY polysaccharides

The antibody response induced against the meningococcal polysaccharides was measured by ELISA. Ninety six-well Maxisorp plates (Nunc, Thermo Fisher Scientific) were coated with the different meningococcal polysaccharides by adding 100 μL/well of a 5 μg/mL polysaccharide solution in PBS buffer at pH 8.2, followed by incubation overnight at 4 °C. After three washings with PBS buffer pH 7.2 containing 0.05% Tween 20 (Sigma) (TPBS), a blocking step was performed by adding 100 μL of BSA solution at 3% in TPBS and incubating the plates for 1 h at 37 °C. After washing as above, eight two-fold serial dilutions of pre-immune, positive and test sera in TPBS were then performed along each column, plates were incubated for 2 h at 37 °C and washed again. One hundred μL/well of secondary antibody coupled to alkaline phosphatase, appropriately diluted in TPBS (anti-mouse IgG, Sigma–Aldrich) were added and the plates incubated for 1 h at 37 °C. After three more washes, 100 μL/well of a 1 mg/mL solution of paranitrophenyl phosphate (p-NPP) (Sigma) in 0.5 M di-ethanolamine buffer pH 9.6 were added. After 30 min of incubation at r.t. plates were read at 405 nm. Sera titers were expressed as the reciprocal dilution corresponding to a cut-off at OD_405_ = 1.0.

### Intracellular T cell staining

Single cell suspensions were obtained from spleens of each mouse by homogenization through a tissue grinder. Fragments were filtered through a 70 μm nylon mesh (Becton-Dickinson, BD). Red blood cells were lysed and cells cultured in RPMI 1640 (Gibco), supplemented with 10% fetal calf serum (Hyclone), 50 μM β-mercaptoethanol (Sigma), 100 U/ml Penicillin, 100 μg/mL Streptomycin, and 2 mM Glutamine (Invitrogen Life Technologies) (Complete medium). For T-cell cytokine responses, cells were stimulated in the presence of anti-CD28 Ab (1 μg/mL) (BD) and CRM_197_ (10 μg/mL), or with anti-CD28 alone (unstimulated), or with anti-CD28 plus anti-CD3 (0.1 μg/mL) (BD). After 2 hours of stimulation, 5 μg/mL Brefeldin A (Sigma Aldrich) were added for an additional 4 h. Cells were washed, stained with live/dead reagent yellow (Invitrogen), fixed in 2% paraformaldehyde, permeabilized in 0.5% saponin and stained with the following mAbs: PerCP-Cy5.5-conjugated anti-CD3 (BD), V500-conjugated anti-CD4 (BD), PE-TexasRed-conjugated anti-CD8 (Invitrogen), PE-conjugated anti-IFNγ (BD), Alexa700-conjugated anti-TNFα (BD), Alexa488-conjugated anti-IL-4 and anti IL-13 (eBioscience), APC-conjugated anti-IL-2 (BD) and PE-Cy7-conjugated anti-IL-17 (eBioscience). Cells were acquired on a LSRII S.O.S.1 (BD) and analyzed using FlowJo software (TriStar). Values displayed are means of groups of five mice. T-cell responses were calculated by subtracting fluorescence percentages of unstimulated samples from the CRM_197_-stimulated samples. Untreated mice were used as negative controls.

### Statistic

The statistical analysis of ELISA titers against meningococcal polysaccharides was performed using GraphPad Prism 6 software, applying Mann-Whitney non-parametric test.

## Additional Information

**How to cite this article**: Buonsanti, C. *et al*. Novel adjuvant Alum-TLR7 significantly potentiates immune response to glycoconjugate vaccines. *Sci. Rep.* **6**, 29063; doi: 10.1038/srep29063 (2016).

## Figures and Tables

**Table 1:** SBA titers values against *N.meningitidis* serogroup C strain 11 of pooled sera from each experimental group described in Fig. 1.

**Antigen**	**Antigen dose (**μ**g)**	**Adjuvant**	**TLR7 agonist dose (**μ**g)**	**SBA**	
				**Post 1**	**Post2**
PBS				<16	<16
CRM-MenC	0.1	Alum		<16	8192
CRM-MenC	0.1	Alum/TLR7	5	64	32768
CRM-MenC	0.1	Alum/TLR7	10	1024	65536
CRM-MenC	0.1	Alum/TLR7	50	512	131072
CRM-MenC	1	Alum		<16	8192
CRM-MenC	1	Alum/TLR7	5	128	32768
CRM-MenC	1	Alum/TLR7	10	256	32768
CRM-MenC	1	Alum/TLR7	50	1024	131072

**Table 2:** SBA titers values against *N.meningitidis* serogroup C strain 11 of pooled sera from each experimental group described in Fig. 3.

**Antigen**	**Antigen dose (**μ**g)**	**Adjuvant**	**TLR7 agonist dose (**μ**g)**	**SBA**
				**Post 2**
CRM-MenC	0.1	Alum		2048
CRM-MenC	0.1	Alum/TLR7	2.5	32768
CRM-MenC	0.1	Alum/TLR7	5	32768
CRM-MenC	0.1	Alum/TLR7	10	32768

**Table 3:** SBA titers values against *N.meningitidis* serogroup A strain F8238, *N.meningitidis* serogroup C strain 11, *N.meningitidis* serogroup W strain 240070, *N.meningitidis* serogroup Y strain 860800 of pooled sera from each experimental group described in Fig. 4.

**Antigen**	**Adjuvant**	**TLR7 agonist dose (**μ**g)**	**Bacterial strain**	**SBA**	
				**Post1**	**Post 2**
	Alum/TLR7	50	MenA	<16	<16
CRM-MenACWY	Alum	0	MenA	<16	<16
CRM-MenACWY	Alum/TLR7	10	MenA	512	4096
CRM-MenACWY	Alum/TLR7	50	MenA	512	>8192
CRM-MenACWY	Alum/TLR7	100	MenA	512	8192
	Alum/TLR7	50	MenC	<16	<16
CRM-MenACWY	Alum	0	MenC	<16	256
CRM-MenACWY	Alum/TLR7	10	MenC	256	4096
CRM-MenACWY	Alum/TLR7	50	MenC	512	16384
CRM-MenACWY	Alum/TLR7	100	MenC	256	4096
	Alum/TLR7	50	MenW	<16	<16
CRM-MenACWY	Alum	0	MenW	<16	128
CRM-MenACWY	Alum/TLR7	10	MenW	<16	1024
CRM-MenACWY	Alum/TLR7	50	MenW	256	4096
CRM-MenACWY	Alum/TLR7	100	MenW	256	1024
	Alum/TLR7	50	MenY	<16	<16
CRM-MenACWY	Alum	0	MenY	128	1024
CRM-MenACWY	Alum/TLR7	10	MenY	512	2048
CRM-MenACWY	Alum/TLR7	50	MenY	1024	8192
CRM-MenACWY	Alum/TLR7	100	MenY	2048	4096

**Table 4:** SBA titers values against *N.meningitidis* serogroup C strain 11 of pooled sera from each experimental group described in Fig. 5.

**Previous immunization**	**Antigen**	**Antigen dose (**μ**g)**	**Adjuvant**	**TLR7 agonist dose (**μ**g)**	**SBA**	
					**Post 1**	**Post2**
–					<16	<16
–	CRM-MenC	1	Alum		128	2048
–	CRM-MenC	1	Alum/TLR7	10	2048	32768
TdaP					<16	<16
TdaP	CRM-MenC	1	Alum		<16	4096
TdaP	CRM-MenC	1	Alum/TLR7	10	1024	16384
